# A novel roseosiphovirus infecting *dinoroseobacter shibae* DFL12^T^ represents a new genus

**DOI:** 10.1186/s12864-025-11274-w

**Published:** 2025-02-08

**Authors:** Nana Wei, Longfei Lu, Yingying Li, Bo Ding, Lanlan Cai, Yunlan Yang

**Affiliations:** 1 State Key Laboratory of Marine Environmental Science, College of Ocean and Earth Sciences, Xiamen University, Xiamen, 361102 China; 2 Archaeal Biology Center, Synthetic Biology Research Center, Shenzhen Key Laboratory of Marine Microbiome Engineering, Key Laboratory of Marine Microbiome Engineering of Guangdong Higher Education Institutes, Institute for Advanced Study, Shenzhen University, Shenzhen, 518055 China; 3 Key Laboratory of Tropical Marine Ecosystem and Bioresource, Fourth Institute of Oceanography, Ministry of Natural Resources, Beihai, 536000 China; 4 Earth, Ocean and Atmospheric Sciences Thrust, The Hong Kong University of Science and Technology (Guangzhou), Guangzhou, China

**Keywords:** Roseophage, Biological features, Genomic analysis, Taxonomy

## Abstract

Bacteria belonging to the *Roseobacter* clade are key players in marine ecosystems, contributing significantly to carbon and sulfur cycles. Marine viruses, particularly those targeting *Roseobacter*, play crucial roles in regulating microbial communities and biogeochemical processes. Despite their importance, phages infecting organisms of the *Roseobacter* clade remain poorly understood. In this study, a novel roseophage, vB_DshS-R26L (R26L), infecting *Dinoroseobacter shibae* DFL12^T^, was isolated and characterized in terms of physiological and genomic properties. R26L has siphovirus morphology with an elongated head and a long, non-flexible tail. The phage has a narrow host range and demonstrates a long infection cycle with a latent period of 3.5 h and a burst size of 22 plaque-forming units (PFU cell^− 1^). R26L possesses a circular, double-stranded DNA genome of 79,534 bp with a G + C content of 62.6%, encoding a total of 116 open reading frames. Notably, seven auxiliary metabolic genes (AMGs), including those related to phosphate metabolism and queuosine biosynthesis, were identified. Phylogenetic and taxonomic analyses revealed that R26L represents a new genus, with its highest intergenomic similarities being 54.7% to another roseophage (R5C). By elucidating the unique characteristics of R26L, this study highlights the complexity of phage infections and the genomic diversity of roseophages, offering valuable insights into the ecological significance of *Roseobacter*–phage interactions in marine environments.

**Supplementary Information:**

The online version contains supplementary material available at 10.1186/s12864-025-11274-w.

## Introduction

With an estimated global number of 10^31^, viruses are widely distributed and play a significant role in shaping microbial communities and driving marine biogeochemical cycles and energy flow in the ocean [1–3]. Additionally, viruses exhibit high recombination rates and frequently exchange genetic material with hosts, representing the largest reservoir of genetic diversity [4]. With the rapid advancement of sequencing technologies, an increasing amount of viral genetic information has been obtained. However, a large number of unknown sequences remain [5]. The isolation and the detailed physiological analysis of viruses continue to be effective methods for deciphering the viral dark matters in environmental samples and revealing new insights into specific virus–host systems [6].

The *Roseobacter* clade, which belongs to the family *Rhodobacteraceae* in the class Alphaproteobacteria, is widely distributed in marine environments, making up 15 − 25% of the total bacterial communities in ocean ecosystems [7]. *Roseobacter* clade is also a significant component of bacterial communities associated with phytoplankton [8], macroalgae [9], and various marine animals [10, 11], exhibiting both mutualistic [12] and pathogenic [13] lifestyles. They are also prevalent in deep-sea and sediment environments [14]. *Roseobacter* clade is known for its versatile ability to perform anoxygenic photosynthesis, degrade organic sulfur compounds, regulate nitrogen content in the ocean, and mediate major biogeochemical processes [15–17]. As one of the most important bacteria in marine ecosystems, members of *Roseobacter* clade are emerging as model organisms.

Although roseophages are less well understood than roseobacters, they have attracted considerable attention and are considered among the most well-studied marine phages. To date, a total of 69 published phages have been isolated from 25 *Roseobacter* strains, but only a small portion of these phages have been thoroughly characterized and analyzed (Table S1). The limited information on phages hinders the understanding of their biological and ecological significance, as well as the interactions between phages and *Roseobacter* clade [18–22]. *Dinoroseobacter shibae* DFL12^T^, a well-studied member of the *Roseobacter* clade [23], is known for its adaptation to extreme salinity and nitrogen limitation, as well as its utilization of light as a crucial resource for survival under starvation conditions [24, 25].

In this study, we report the isolation and identification of vB_DshS-R26L (hereafter referred to as R26L), a novel phage that infects *D. shibae* DFL12^T^. Through physiological and genomic analyses, we delineate the characteristics of R26L and its evolutionary relationship with previously known phages.

## Materials and methods

### Phage isolation, purification and amplification

Surface water samples were collected from the Pearl River Estuary, China, and filtered through 0.22 μm filters (Millipore, Bedford, MA, USA). The samples were stored at 4 °C in the dark. *D. shibae* DFL12^T^ was cultured as the host in 2216E medium at 28 °C with shaking at 160 rpm min^− 1^. The filtered seawater was then incubated with the host for 24 h to enrich the phage population. Subsequently, the cell culture was filtered through a 0.22 μm membrane and used for phage isolation employing the double-layer agar method [26]. Following five rounds of purification, the phages were propagated in liquid 2216E medium. Phages were collected by centrifugation (10,000× g, 4 °C) and filtration (0.22 μm). To achieve high phage titers, the filtrate was supplemented with 10% polyethylene glycol 8,000 to precipitate virions, followed by incubation at 4 °C for 12 h. Phages were then harvested by centrifugation at 10,000× g for 1 h and resuspended in SM buffer [27]. For further concentration, CsCl equilibrium gradient centrifugation was employed at 200,000× g at 4 °C for 12 h [28]. The collected phages were desalted using SM buffer and stored at 4 °C for subsequent experiments.

### Morphological analysis by transmission electron microscopy (TEM)

The morphology of the phage was analyzed by TEM. Approximately 10 µL of phage solution with a titer of 10^7^ PFU mL^− 1^ was applied to 200-mesh carbon-coated copper grids, allowed to adsorb in the dark for 30 min, negatively stained with 1% phosphotungstic acid, and air-dried. Samples were observed with a JEM-2100 TEM (JEOL, Tokyo, Japan), and phage images were processed using ImageJ software [29].

### Host range analysis

To determine the host range, a total of 17 bacterial strains (Table 1) were cultured in 2216E media at 37 °C for 12 h. For the spot assay, 1 mL of each bacterial culture (OD_600_: 0.3 ~ 0.6) was mixed with 5 mL of soft agar (0.5%, maintained at 45 °C) and immediately poured onto plates containing 1.5% agar. Serially diluted phage lysates (10^5^, 10^6^, 10^7^, and 10^8^ PFU mL^− 1^) were then spotted (5 µL) onto the prepared plates, which were incubated at 28 °C. The infectivity was assessed based on the presence of plaques.

**Table 1:** Host range of vB_DshS-R26L (+, infected; −, uninfected)

Species	R26L	R5C
*Dinoroseobacter shibae* DFL12^T^	−	−
*Roseovarius crassostreae* CV919-312^T^	−	NA
*Dinoroseobacter* sp. JL1447	−	−
*Roseovarius aestuarii* SMK-122^T^	−	NA
*Ruegeria mobilis* NBRC 101,030^T^	−	NA
*Roseovarius crassostreae* CV919-312^T^	−	NA
*Erythrobacter litoralis* DSM 8509	−	−
*Roseobacter denitrificans* OCh114	−	−
*Erythrobacter longus* DSM 6997^T^	−	NA
*Roseovarius indicus* JL4234	−	−
*Roseovarius halotolerans* HJ50^T^	−	−
*Roseovarius indicus* JL4258	−	NA
*Roseomonas cervicalis* ATCC 49,957^T^	−	−
*Erythrobacter flavus* SW-46^T^	−	−
*Roseovarius crassostreae* CV919-312^T^	−	NA
*Paenibacillus glucanolyticus* DSM 5162^T^	−	−
*Roseovarius nanhaiticus* NH52J^T^	−	NA

NA, Not Available

### One-step growth curve

The infectivity and replication ability of the phage were analyzed using one-step growth curve experiment [30]. In brief, phages were mixed with 1 mL of host cells in the exponential growth phase at a multiplicity of infection (MOI) of 0.01 and incubated at 37 °C for 15 min to allow adsorption. Unabsorbed phages were removed by centrifugation (10,000× g at 4 °C for 5 min), and the pellets infected by phages were resuspended in 50 mL of fresh 2216E medium, then incubated at 28 °C with agitation at 160 rpm min^− 1^. Phage titers were determined hourly using the double-layer agar method. The latent period refers to the time from the start of culture to the onset of phage release; the burst size is calculated as the ratio of plaques formed after phage burst to the initial plaque count.

### DNA extraction and sequencing

The high titer phages were treated with protease K (100 mg mL^− 1^), sodium dodecyl sulfate (10% [wt/vol]), and EDTA (0.5 mol mL^− 1^; pH 8.0). After 3 h of digestion at 55 °C, phage DNA was extracted using the phenol-chloroform method [27]. DNA from the supernatant was sequentially precipitated with isopropanol and stored at -20 °C overnight. The precipitate was washed twice with 70% ethanol before air drying and finally dissolved in sterile Tris-EDTA buffer (10 mM Tris-HCl and 1 mM EDTA [pH 8.0]). The DNA concentration was determined using a Qubit dsDNA BR kit and a Qubit fluorometer (ThermoFisher Scientific, Waltham, MA). Subsequently, the extracted phage genomic DNA was sequenced on the Illumina HiSeq 4000 platform with a 150-bp paired-end DNA library. The high-quality reads were then assembled *de novo* using Newbler assembler version 2.8 to generate the final assembled sequence.

### Genome annotation and comparative genomic analysis

Phage termini and packaging mechanisms were predicted using PhageTerm (v3.0.1) [31]. Putative open reading frames (ORFs) were identified using the online GeneMarkS server (v4.32) and putative tRNA genes were detected using tRNAscan-SE v2.0 [32, 33]. ORFs were further annotated by BLASTP and the NCBI conserved domains database, with a cutoff E-value of 10^− 3^, as well as Pfam (parameters: E-value < 0.01) and HHPred (parameters: E-value < 0.001, cols > 80) [34–36]. A circular genome map was generated using Proksee (https://proksee.ca), and phage comparisons were conducted using Clinker [37]. The raw Illumina sequencing data produced in this study have been deposited in the NCBI Sequence Read Archive (SRA) under the BioProject accession number PRJNA1192592, and the complete phage genome sequence has been deposited in the NCBI GenBank database under accession number PP882867.

### Taxonomic network and phylogenetic analysis

Viral CONTigs Automatic Clustering and Taxonomy v.2.0 (vConTACT2) was employed to assess the similarity between the isolated phage and other phages in the Prokaryotic Viral RefSeq 207 database based on whole-genome gene-sharing profiles, retaining only results with similarity scores of ≥ 1 [38]. The network graph was visualized using Cytoscape (version 3.8.0), with edge-weighted spring-embedded modeling based on vConTACT2 output similarity scores [39]. The arrangement of phage genomes within the network was determined by the number of shared protein clusters. To determine the phylogenetic relationships of phages, the predicted phage genomes from vConTACT2 results and the genomes of published roseophages were uploaded to the VICTOR server (https://ggdc.dsmz.de/victor.php) for tree construction [40]. All pairwise comparisons of viral amino acid sequences were conducted using the genome BLAST distance phylogeny (GBDP) method, following the recommended settings for prokaryotic viruses [41]. The resulting phylogenetic tree was visualized and manipulated using tvBOT in this study [42].

## Results

### Biological features of R26L

**Fig. 1:**
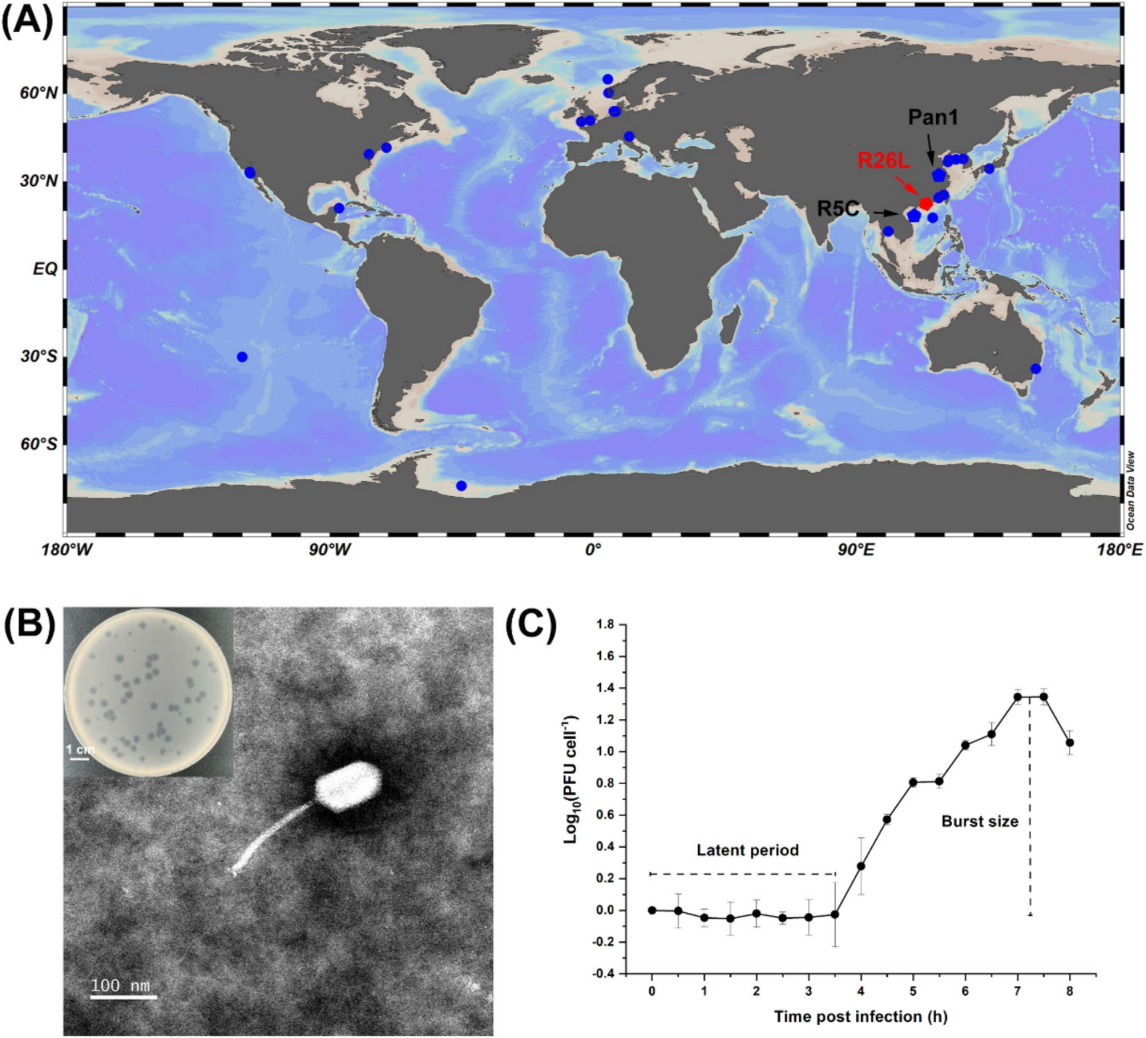
Biological features of phage R26L. (**A**) A map displaying the sampling sites of roseophages, with a red dot representing R26L and blue dots indicating other published roseophages. The map was generated using Ocean Data View (version 5.3.0). (**B**) Transmission electron microscopy image of R26L. Scale bar, 100 nm. Insert shows plaques formed by phage R26L infection of *D. shibae* DFL12^T^ with a scale bar of 1 cm. (**C**) One-step growth curve of phage R26L showing the latent period and burst size. Phages were incubated with an early log-phase *D. shibae* DFL12^T^ at a multiplicity of infection of 0.01. Each data point is shown as the mean values and standard deviations of three independent replicates

The phage infecting *D. shibae* DFL12^T^ was isolated from seawater collected from the Pearl River Estuary, China (113.72°E, 22.22°N) (Fig. 1A), and designated as vB_DshS-R26L (R26L) [43]. The plaques formed by R26L were circular, with clear centers and blurred edges, measuring approximately 1 to 3.5 mm in diameter after 48–72 h of incubation at 28 °C (Fig. 1B). TEM analysis revealed that R26L is siphovirus-like, characterized by a long, non-flexible tail measuring 170.35 ± 2.82 nm and an elongated head of 104.85 ± 3.29 nm (Fig. 1B).

Host infectivity testing demonstrated that R26L failed to infect any tested *Roseobacter* strains beyond its original host *D. shibae* DFL12^T^ (Table 1), thus is characterized by a narrow host range. The one-step growth curve indicated that R26L required approximately seven hours to complete one infection cycle, and the latent period was about 3.5 h, followed by a burst phase, resulting in the release of approximately 22 PFU cell^− 1^ (Fig. 1C).

### Genomic features of R26L

**Fig. 2:**
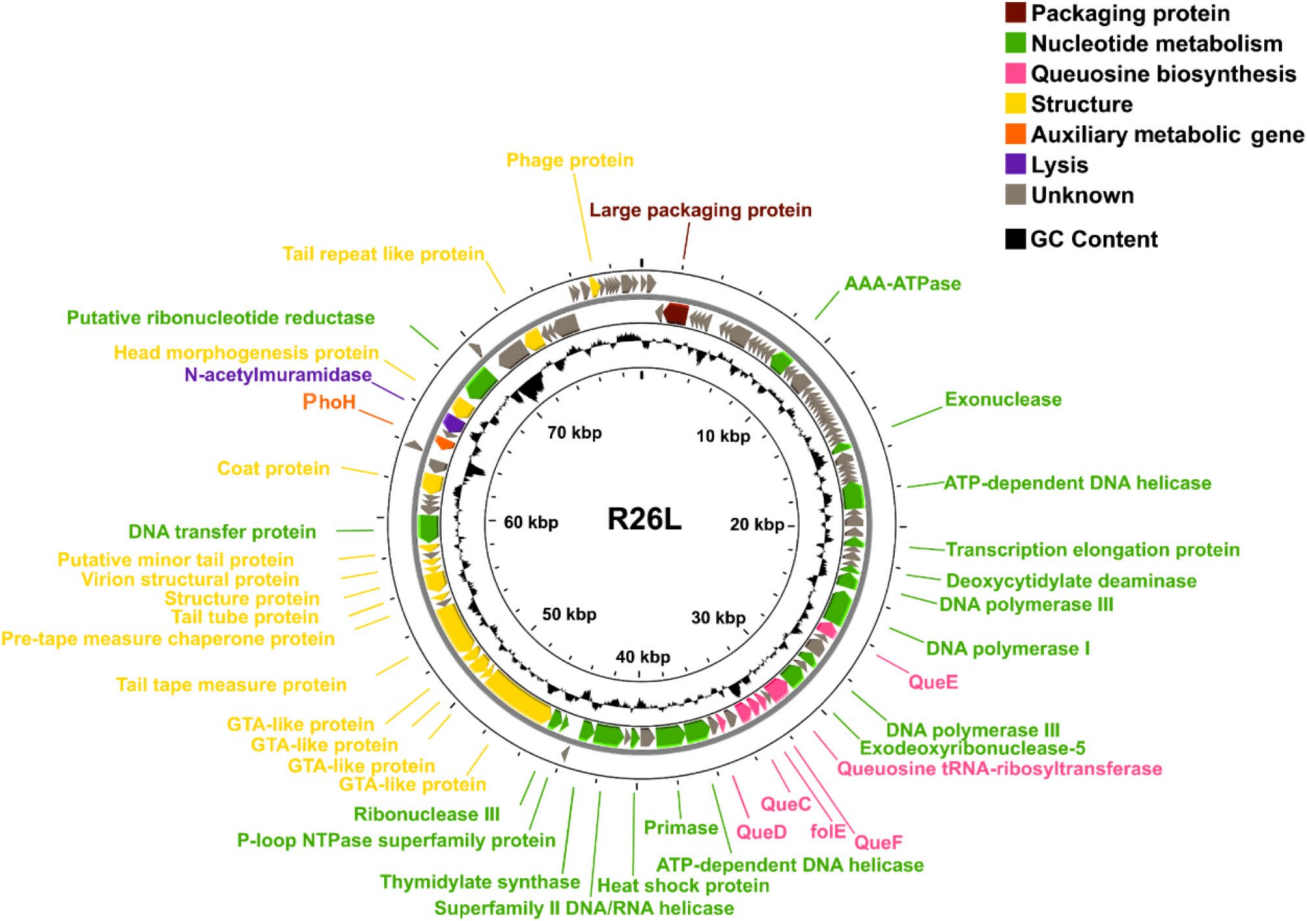
The genome map of R26L. The orientation of each ORF corresponds to the direction of transcription and the predicted functions of the proteins encoded by the genes are illustrated in different colors (outer ring). GC content plots are generated by calculating the GC content for each sliding window (inner ring)

The sequencing of phage R26L produced a complete contig of 79,534 bp, with a circular double-stranded DNA genome having a G + C content of 62.6%, slightly lower than that of its host (63.1%) [23, 44]. The properties of the genome, including the positions, orientations, and putative functions of each gene, were detailed in the supplementary files (Table S2). We predicted a total of 116 ORFs, of which 42 were predicted to have specific functions (Fig. 2). Based on their functions, the ORFs were classified into seven categories, relating to nucleotide metabolism, structure, packaging, queuosine biosynthesis, lysis, auxiliary metabolic genes (AMGs) and unknown functions. Eighteen genes associated with nucleotide metabolism, constituting 25.7% of the genome, form a nucleic acid metabolism module that includes genes encoding DNA helicase, DNA polymerase, primase, thymidylate synthase, etc. Fourteen genes were related to structural components, five were involved in queuosine biosynthesis, and one gene, classified as an AMG, was also identified as the phosphate starvation-inducible gene *phoH*. R26L also encodes a lysozyme, *N*-acetylmuramidase, known for facilitating the lysis of the host cell wall in phage N4 [45, 46]. No lysogeny-related markers, such as transposase or integrase, excisionase, and repressor, were detected. Additionally, one tRNA gene encoding tryptophan (*trp*) was identified in R26L.

### Phylogeny and taxonomy of R26L

**Fig. 3:**
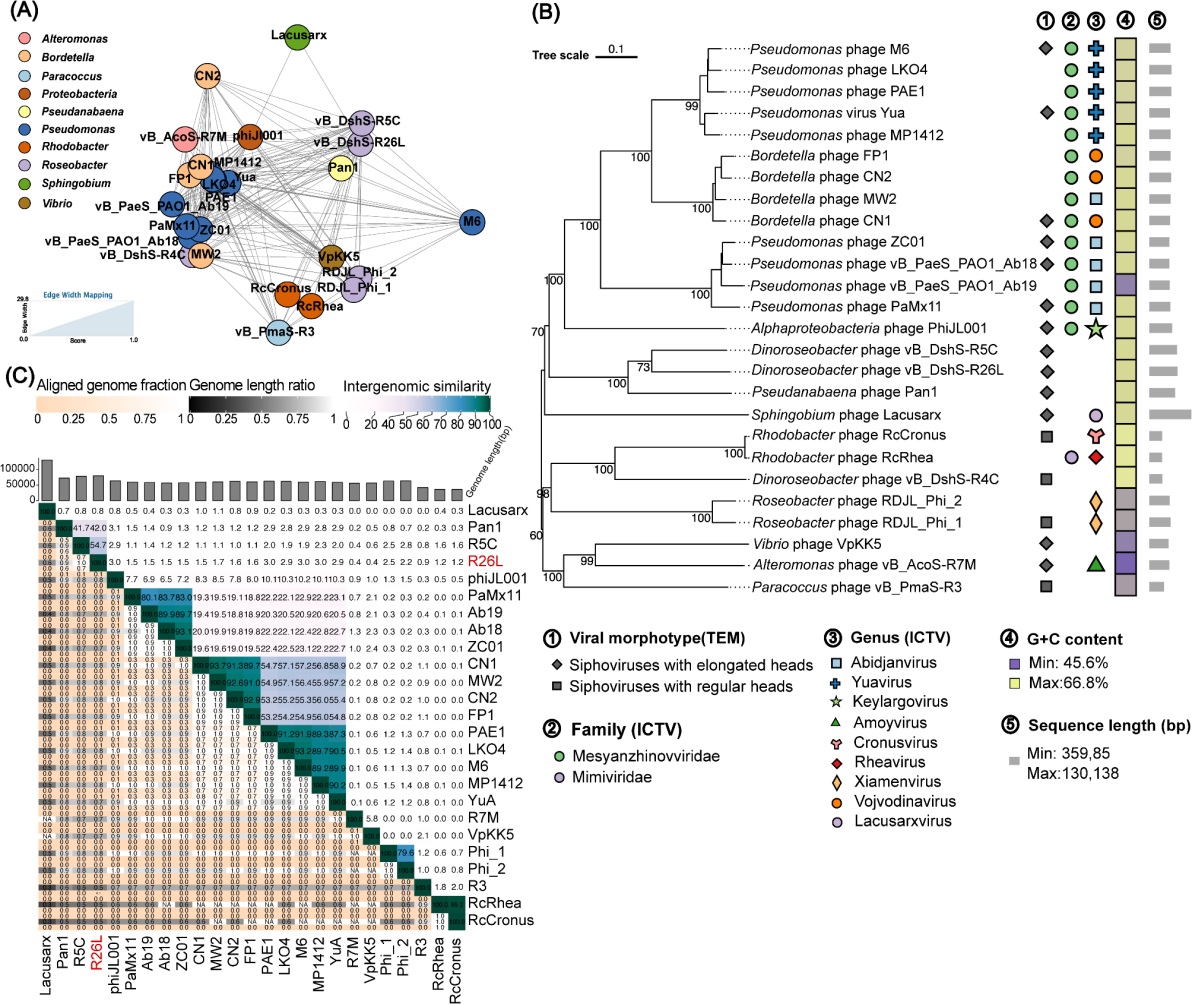
Taxonomic and phylogenetic analysis of R26L. (**A**) Protein-sharing network indicating evolutionary affinity among vB_DshS-R26L and its related phages sharing pairwise similarity scores of > 1. Each node represents a phage genome and is colored according to its host taxonomy. Edges connecting pairwise phages from the same viral cluster determined by vConTACT2 are displayed. Thicker edges indicate a strong connection between the two phages. The valid names of existing host genera for the phages are displayed. (**B**) GBDP tree based on complete or partial genomes of compared phages by the web tool, VICTOR. Viral morphotypes are marked according to their published TEM pictures, and the family and genus cluster information was obtained from the International Committee on Taxonomy of Viruses (ICTV). (**C**) Pairwise intergenomic distances/similarities among viral genomes for 25 phages as per the Virus Intergenomic Distance Calculator

On the basis of NCBI BLASTN analysis, *Dinoroseobacter* phage vB_DshS-R5C (R5C) and *Pseudanabaena* phage Pan1 shared sequence identities of 92.4% and 76.2% with R26L, respectively, but with query coverages of only 43% and 31%. To further understand the relationship between R26L and known phages and to determine its taxonomy, a database comparison, phylogenetic analysis, and intergenomic similarity assessment were conducted in this study. A total of 25 phages, with significant similarity scores > 1 as calculated using vConTACT2, were identified in the Prokaryotic Viral RefSeq 207 databases. These phages were isolated against hosts including *Roseobacter*, *Pseudanabaena*, *Rhodobacter*, and *Sphingobium*, with phages R5C and Pan1 showing the highest similarity scores of 169.30 and 116.61 with R26L, respectively (Table S3, Fig. 3A). Phylogenetic analysis revealed that, although R26L clustered with R5C and Pan1, their genetic distance was sufficient to warrant their classification as distinct phages (Fig. 3B). Furthermore, VIRIDIC analysis revealed that intergenomic similarity between R26L and these phages ranged from 0.8 to 54.7%, with R5C and Pan1 showing the highest similarities at 54.7% and 42.0%, respectively, still below the 70% threshold for genus classification (Fig. 3C) [47]. Overall, R26L exhibits distinctive genomic features that set it apart from other known phages, supporting it to be a new, unclassified genus.

### Comparative genomic analysis

**Fig. 4:**
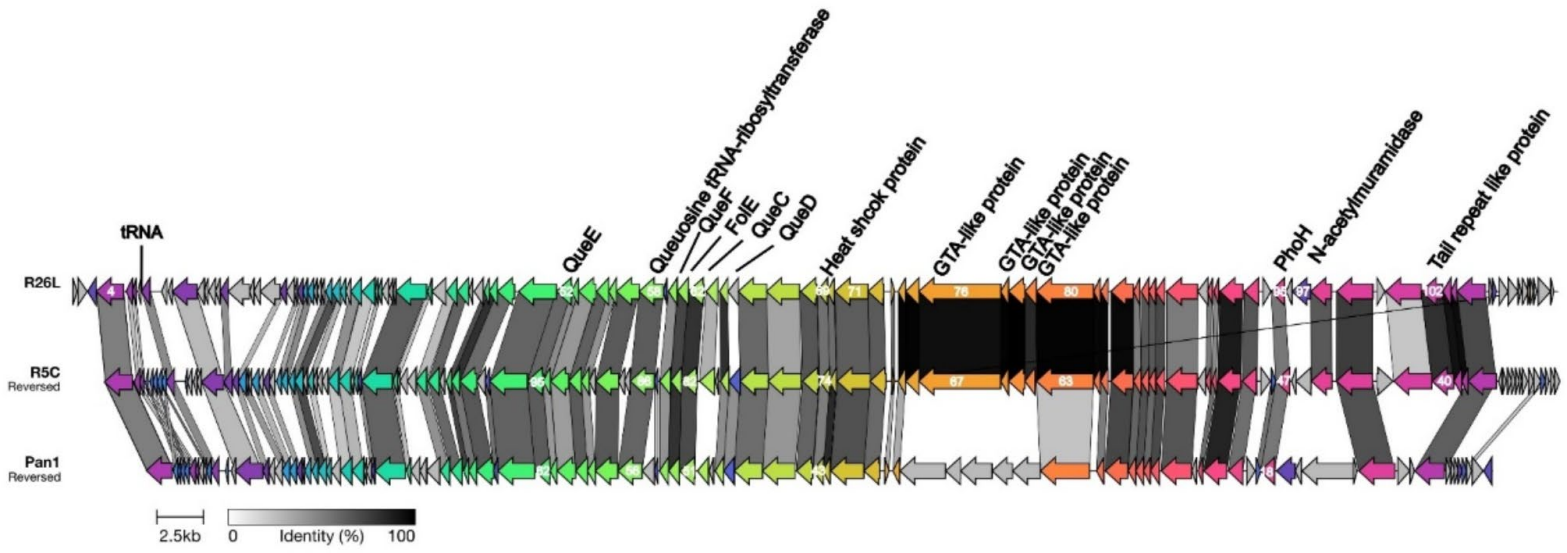
Genome organization and comparison of the phage R26L to R5C and Pan1. Arrows indicate the direction of transcription of each gene and the color indicates different ORFs, with arrows of the same color representing related ORFs. Key genes and their corresponding gene numbers are labeled on the arrows for clarity. The gradient shades of grey in the bar represent the percentage of amino acid sequence identity between the two phages

A comparative analysis of gene function was conducted among R26L, R5C, and Pan1 to highlight both functional differences and similarities, offering insights into shared and unique genetic features, particularly in key areas such as structural proteins, nucleic acid metabolism, and other functional modules (Fig. 4). Phage R26L shares 72 (62.1%) ORFs with R5C and 60 ORFs (51.7%) with Pan1, with sequence similarity ranging from 32.1 to 99.7% for R26L–R5C and 31.8–86.1% for R26L–Pan1 (Table S4 and S5). Most of the genes associated with protein structure in phages R26L and R5C exhibit a high degree of sequence similarity. However, certain structural proteins (gp76 − 79) in R26L and R5C show no similarity to those in Pan1. For instance, both R26L and R5C possess additional tail structure genes (gp101 − 104) that are absent in Pan1, possibly due to their shared host origin. While R26L encodes a greater number of nucleic acid metabolism-related genes than R5C and Pan1, the genes involved in nucleic acid metabolism show comparable levels of similarity across all three phages.

The *phoH* gene is highly conserved in R26L, R5C and Pan1, with amino acid identity > 60% (calculated by BLASTp). All three phages possess queuosine biosynthesis genes (*queC*, *queD*, *queE*, *queF*, and *folE*), which are thought to modify viral DNA, protecting it from the host immune system [48]. R26L harbors four gene transfer agents (GTA) proteins (gp76 − 79), which exhibit high homology to the corresponding proteins in R5C (gp64 − 67), with sequence identities ranging from 95.1 to 100%. Both R26L and R5C have heat shock genes, which play critical roles in protein folding and stability [26, 49]. Unlike phage R26L, both phages R5C and Pan1 lack tRNA genes, and none of the three phages carry lysogenic genes.

## Discussion

The *Roseobacter* clade represents a crucial part of the microbial community within marine ecosystems, playing key roles in the biogeochemical cycling of carbon, sulfur, and nitrogen [16]. Roseophages not only regulate microbial community dynamics through host lysis but also influence host evolution and metabolism via horizontal gene transfer (HGT) [26]. Therefore, studying roseophages is essential for a comprehensive understanding of these processes. This study focuses on a newly isolated phage R26L, which infects *D. shibae* DFL12^T^, revealing its physiological and genomic features. After R26L lysed *D. shibae* DFL12^T^ on the plate, the surrounding bacterial density decreased, leading to the formation of circular plaques with clearly defined edges. Simultaneously, no obvious halo was observed around the plaques, indicating a reduction or absence of bacterial polysaccharide capsules surrounding the phage plaques [50]. Among the published roseophages, only approximately 25% have had their plaque morphologies characterized, and only four phages (R4C, R5C, VP1, and PM1) have been documented with plaque photographs (Table S1). Similar to R26L, none of these plaques were reported to exhibit obvious halos [51].

A review of phages’ one-step growth curves over the past seven decades shows that the burst size of phages ranges from 10 to 1000 PFU cell^− 1^ [52]. The burst size of R26L is near the known minimum of phages, suggesting poor codon adaptation to the host translation machinery and a limited ability to utilize the host’s tRNA inventory [53]. While R26L containing a tRNA may offer some advantage by supplementing the host’s tRNA pool, it might not be enough to significantly improve viral replication or resource utilization. In addition, the latent period of R26L is extended by two hours compared to R5C, and its burst size is also lower than that of R5C (65 PFU per cell^− 1^) [54]. Notably, R26L ranks fourth in terms of genome size among all roseophages (Table S1), with the largest phage genome isolated from host *D. shibae* DFL12^T^. A larger genome size indicated that the virus might have a more intricate life cycle, more functional genes, or more regulatory elements, which could be associated with enhanced host adaptability, host range, or complex interactions with the environment [55]. Moreover, other factors, such as adsorption efficiency, differences in encoded proteins, and environmental conditions, may also play significant roles in influencing burst size. As definitive evidence is currently lacking, further investigation into differences in gene function expression during infection could provide valuable insights into this phenomenon.

R26L likely exhibits a high degree of specificity for its original host, potentially attributed to evolutionary constraints imposed by specific host environments that limit its adaptability to alternative hosts [56]. A similarly narrow host range has also been observed in roseophages such as DSS3Φ8, RDJLΦ1, and R5C, which possess long and flexible tails [26, 57, 58]. The tail structure is essential for phage host specificity and infection, as phages use receptor-binding proteins (RBPs) on tail fibers or baseplate proteins to recognize and bind to host cell surface receptors. This interaction triggers conformational changes in the tail, forming a channel through which the phage injects its DNA into the host cell [59].

AMGs are frequently identified in viral genomes and estimated to be capable of regulating host metabolism or stress response during infection [60–62]. R26L is annotated with seven AMGs, while the known roseophages, on average, carry 3.75 AMGs [49]. The AMGs of R26L include the *phoH* gene and queuosine biosynthesis genes (*queC*, *queD*, *queE*, *folE*, *queF*). The *phoH* gene, the protein product of which belongs to *pho* regulon, is widely distributed in prokaryotes and viruses [63] and has been widely used as an effective biomarker gene [64]. Some phosphorus acquisition genes, such as *phoH* and *pstS*, and alkaline phosphatase synthesis gene *phoA* exist in the phage genomes, which are beneficial to host cells to enhance phosphorus uptake and transport under a low phosphorus environment during infection for efficient phage replication and production [65, 66]. Several *phoH* from α-proteobacteria were placed in the cyanobacterial cluster, suggesting potential HGT of *phoH* genes between these phyla, possibly mediated by phages [67]. As an essential precursor for the synthesis of queuosine in bacteria [48], preQ_0_ was catalyzed by four enzymes: FolE, QueD, QueC, and QueE. In bacteria, preQ_0_ is reduced to 7-aminomethyl-7-deazaguanine (preQ_1_) by QueF [68–70] before tRNA-guanine-transglycosylases incorporate it in tRNA [71]. R26L was predicted to modify its DNA with queuosine to protect phage DNA from host restriction systems, distinct from phage vB_AcoS-R7M and phage 9 g [27, 72]. Both R26L and R5C were annotated with genes encoding GTA, indicating that *phoH* and queuosine biosynthesis genes may have been introduced by their common ancestor through HGT in early evolution and maintained due to positive selection.

GTAs are phage-like particles that influence bacterial diversity and adaptation across various ecological niches [73]. Roseophages may act as vehicles for HGT, facilitating the dissemination of adaptive traits such as antibiotic resistance genes and virulence factors among bacterial communities [74]. Comparative genomic studies have shown that GTA genes are highly conserved among related roseophages, highlighting their evolutionary significance in phage-mediated HGT processes [75]. This cross-domain genomic similarity highlights the evolutionary flexibility of these phages and their capacity for significant genetic exchange across different microbial domains. Phages continuously evolve to develop unique functions, leading to the formation of distinct groups from known phages. These findings reveal the complex evolutionary dynamics and adaptive strategies employed by these viruses, providing valuable insights into their ecological roles and interactions with diverse hosts.

## Conclusion

This study successfully isolated and comprehensively characterized the roseophage, vB_DshS-R26L. While R26L and R5C share the same host, exhibit similar morphology, and have comparable genetic structures, they differ significantly in terms of their infection processes (e.g., latent period and burst size) and intergenomic similarity, which suggests the classification of R26L as a new genus. Moreover, R26L contains several AMGs involved in phage–host interactions, including the phosphate metabolism-related *phoH* gene and queuosine biosynthesis genes. Its genetic signature as a vehicle for horizontal gene transfer highlights its role in driving evolutionary processes and diversity, offering new insights into the interactions between *Roseobacter* and their phages.

## Electronic supplementary material

Below is the link to the electronic supplementary material.

Supplementary Material 1

## Data Availability

The raw Illumina sequencing data in this study have been deposited in the NCBI Sequence Read Archive (SRA) under the BioProject accession number PRJNA1192592, and the genome sequence of vB_DshS-R26L has been deposited in the GenBank database under the accession number PP882867.

## Abbreviations

ORFs: Putative open reading frames
PFU: Plaque-forming units
MOI: Multiplicity of infection
GBDP: Genome BLAST distance phylogeny
AMGs: Auxiliary metabolic genes
Trp: Tryptophan
GTA: Gene transfer agents
HGT: Horizontal gene transfer
RBPs: Receptor binding proteins

## References

[1] Suttle CA. Viruses in the sea. Nature. 2005;437(7057):356–61. 16163346 10.1038/nature04160

[2] Suttle CA. Marine viruses — major players in the global ecosystem. Nat Rev Microbiol. 2007;5(10):801–12. 17853907 10.1038/nrmicro1750

[3] Zimmerman AE, Howard-Varona C, Needham DM, John SG, Worden AZ, Sullivan MB, et al. Metabolic and biogeochemical consequences of viral infection in aquatic ecosystems. Nat Rev Microbiol. 2020;18(1):21–34. 31690825 10.1038/s41579-019-0270-x

[4] Brum JR, Ignacio-Espinoza JC, Roux S, Doulcier G, Acinas SG, Alberti A, et al. Patterns and ecological drivers of ocean viral communities. Science. 2005;7(9):1393–405. 10.1126/science.1261498 25999515

[5] Roux S, Hallam SJ, Woyke T, Sullivan MB. Viral dark matter and virus–host interactions resolved from publicly available microbial genomes. eLife. 2015;4:e08490. 26200428 10.7554/eLife.08490 PMC4533152

[6] Chevallereau A, Pons BJ, Van Houte S, Westra ER. Interactions between bacterial and phage communities in natural environments. Nat Rev Microbiol. 2022;20(1):49–62. 34373631 10.1038/s41579-021-00602-y

[7] Kent AG, Garcia CA, Martiny AC. Increased biofilm formation due to high-temperature adaptation in marine *Roseobacter*. Nat Microbiol. 2018;3(9):989–95. 30061756 10.1038/s41564-018-0213-8 PMC6119078

[8] Amin SA, Parker MS, Armbrust EV. Interactions between diatoms and bacteria. Microbiol Mol Biol Rev. 2012;76(3):667–84. 22933565 10.1128/MMBR.00007-12 PMC3429620

[9] Ashen JB, Goff LJ. Molecular and ecological evidence for species specificity and coevolution in a group of marine algal-bacterial symbioses. Appl Environ Microbiol. 2000;66(7):3024–30. 10877801 10.1128/aem.66.7.3024-3030.2000 PMC92106

[10] Apprill A, Marlow HQ, Martindale MQ, Rappé MS. The onset of microbial associations in the coral *Pocillopora meandrina*. ISME J. 2009;3(6):685–99. 19242535 10.1038/ismej.2009.3

[11] Barbieri E, Paster BJ, Hughes D, Zurek L, Moser DP, Teske A, et al. Phylogenetic characterization of epibiotic bacteria in the accessory nidamental gland and egg capsules of the squid *Loligo pealei* (Cephalopoda: Loliginidae). Environ Microbiol. 2001;3(3):151–67. 11321532 10.1046/j.1462-2920.2001.00172.x

[12] Geng H, Belas R. Molecular mechanisms underlying roseobacter–phytoplankton symbioses. Curr Opin Biotech. 2010;21(3):332–38. 20399092 10.1016/j.copbio.2010.03.013

[13] Fernandes N, Case RJ, Longford SR, Seyedsayamdost MR, Steinberg PD, Kjelleberg S, et al. Genomes and virulence factors of novel bacterial pathogens causing bleaching disease in the marine red alga *Delisea Pulchra*. PLoS ONE. 2011;6(12):e27387. 22162749 10.1371/journal.pone.0027387 PMC3230580

[14] Brinkhoff T, Giebel H-A, Simon M. Diversity, ecology, and genomics of the *Roseobacter* clade: a short overview. Arch Microbiol. 2008;189(6):531–39. 18253713 10.1007/s00203-008-0353-y

[15] Luo H, Moran MA. Evolutionary ecology of the marine *Roseobacter* clade. Microbiol Mol Biol Rev. 2014;78(4):573–87. 25428935 10.1128/MMBR.00020-14 PMC4248658

[16] Buchan A, González JM, Moran MA. Overview of the marine *Roseobacter* lineage. Appl Environ Microb. 2005;71:344–53. 10.1128/AEM.71.10.5665-5677.2005 PMC1265941 16204474

[17] Liu L, Chen X, Ye J, Ma X, Han Y, He Y, et al. Sulfoquinovose is a widespread organosulfur substrate for *Roseobacter* clade bacteria in the ocean. ISME J. 2023;17(3):393–405. 36593260 10.1038/s41396-022-01353-1 PMC9938184

[18] Bischoff V, Bunk B, Meier-Kolthoff JP, Spröer C, Poehlein A, Dogs M, et al. Cobaviruses– a new globally distributed phage group infecting *Rhodobacteraceae* in Marine ecosystems. ISME J. 2019;13:1404–21. 30718806 10.1038/s41396-019-0362-7 PMC6775973

[19] Zhan Y, Chen F. Bacteriophages that infect marine *roseobacters*: genomics and ecology. Environ Microbiol. 2019;21(6):1885–95. 30556267 10.1111/1462-2920.14504

[20] Du S, Wu Y, Ying H, Wu Z, Yang M, Chen F et al. Genome sequences of the first *Autographiviridae* phages infecting marine *Roseobacter*. Microb Genom. 2024; 2024;10:001240. 10.1099/mgen.0.001240 PMC11092137 38630615

[21] Wu Z, Guo L, Wu Y, Yang M, Du S, Shao J, et al. Novel phage infecting the *Roseobacter* CHUG lineage reveals a diverse and globally distributed phage family. mSphere. 2024;9(7):e00458–24. 38926906 10.1128/msphere.00458-24 PMC11288001

[22] Zhang Z, Wu Z, Liu H, Yang M, Wang R, Zhao Y, et al. Genomic analysis and characterization of phages infecting the marine *Roseobacter* CHAB-I-5 lineage reveal a globally distributed and abundant phage genus. Front Microbiol. 2023;14:1164101. 37138617 10.3389/fmicb.2023.1164101 PMC10149686

[23] Wagner-Döbler I, Ballhausen B, Berger M, Brinkhoff T, Buchholz I, Bunk B, et al. The complete genome sequence of the algal symbiont *Dinoroseobacter shibae*: a hitchhiker’s guide to life in the sea. ISME J. 2010;4(1):61–77. 19741735 10.1038/ismej.2009.94

[24] Kleist S, Ulbrich M, Bill N, Schmidt-Hohagen K, Geffers R, Schomburg D. Dealing with salinity extremes and nitrogen limitation– an unexpected strategy of the marine bacterium *Dinoroseobacter shibae*. Environ Microbiol. 2017;19(3):894–908. 26914854 10.1111/1462-2920.13266

[25] Soora M, Tomasch J, Wang H, Michael V, Petersen J, Engelen B et al. Oxidative stress and starvation in *Dinoroseobacter shibae*: the role of extrachromosomal elements. Front Microbiol. 2015;6. 10.3389/fmicb.2015.00233 PMC4373377 25859246

[26] Yang Y, Cai L, Ma R, Xu Y, Tong Y, Huang Y, et al. A novel Roseosiphophage isolated from the oligotrophic South China Sea. Viruses. 2017;9(5):109. 28505134 10.3390/v9050109 PMC5454422

[27] Ma R, Lai J, Chen X, Wang L, Yang Y, Wei S, et al. A novel phage infecting *Alteromonas* represents a distinct group of siphophages infecting diverse aquatic copiotrophs. mSphere. 2021;6(3):e00454–21. 34106770 10.1128/mSphere.00454-21 PMC8265664

[28] Lawrence JE, Steward GF. Purification of viruses by centrifugation. Man Aquat Viral Ecol. 2010;17:166–81.

[29] Schneider CA, Rasband WS, Eliceiri KW. NIH Image to ImageJ: 25 years of image analysis. Nat Methods. 2012;9:671. 22930834 10.1038/nmeth.2089 PMC5554542

[30] Middelboe M, Chan AM, Bertelsen SK. Isolation and life cycle characterization of lytic viruses infecting heterotrophic bacteria and cyanobacteria. Man Aquat Viral Ecol. 2010;118–33.

[31] Garneau JR, Depardieu F, Fortier L-C, Bikard D, Monot M. PhageTerm: a tool for fast and accurate determination of phage termini and packaging mechanism using next-generation sequencing data. Sci Rep. 2017;7:8292. 28811656 10.1038/s41598-017-07910-5 PMC5557969

[32] Besemer J. GeneMarkS: a self-training method for prediction of gene starts in microbial genomes. Implications for finding sequence motifs in regulatory regions. Nucleic Acids Res. 2001;29(12):2607–18. 11410670 10.1093/nar/29.12.2607 PMC55746

[33] Chan PP, Lin BY, Mak AJ, Lowe TM. tRNAscan-SE 2.0: improved detection and functional classification of transfer RNA genes. Nucleic Acids Res. 2021;49(16):9077–96. 34417604 10.1093/nar/gkab688 PMC8450103

[34] AltschuP SF, Gish W, Miller W, Myers EW, Lipman DJ. Basic local alignment search tool. J Mol Biol. 1990;215:403–10. 2231712 10.1016/S0022-2836(05)80360-2

[35] Zimmermann L, Stephens A, Nam S-Z, Rau D, Kübler J, Lozajic M, et al. A completely reimplemented mpi bioinformatics toolkit with a new hhpred server at its core. J Mol Biol. 2018;430(15):2237–43. 29258817 10.1016/j.jmb.2017.12.007

[36] El-Gebali S, Mistry J, Bateman A, Eddy SR, Luciani A, Potter SC, et al. The Pfam protein families database in 2019. Nucleic Acids Res. 2019;47:D427–32. 30357350 10.1093/nar/gky995 PMC6324024

[37] Gilchrist CLM, Chooi Y-H. Clinker & clustermap.js: automatic generation of gene cluster comparison figures. Bioinformatics. 2021;37(16):2473–75. 33459763 10.1093/bioinformatics/btab007

[38] Jang HB, Bolduc B, Zablocki O, Kuhn JH, Roux S, Adriaenssens EM, et al. Taxonomic assignment of uncultivated prokaryotic virus genomes is enabled by gene-sharing networks. Nat Biotechnol. 2019;37(6):632–39. 31061483 10.1038/s41587-019-0100-8

[39] Shannon P, Markiel A, Ozier O, Baliga NS, Wang JT, Ramage D, et al. Cytoscape: a software environment for integrated models of biomolecular interaction networks. Genome Res. 2003;13(11):2498–504. 14597658 10.1101/gr.1239303 PMC403769

[40] Meier-Kolthoff JP, Göker M. VICTOR: genome-based phylogeny and classification of prokaryotic viruses. Bioinformatics. 2017;33(21):3396–404. 29036289 10.1093/bioinformatics/btx440 PMC5860169

[41] Meier-Kolthoff JP, Auch AF, Klenk H-P, Göker M. Genome sequence-based species delimitation with confidence intervals and improved distance functions. BMC Bioinf. 2013;14:60. 10.1186/1471-2105-14-60 PMC3665452 23432962

[42] Xie J, Chen Y, Cai G, Cai R, Hu Z, Wang H. Tree visualization by one table (tvbot): a web application for visualizing, modifying and annotating phylogenetic trees. Nucleic Acids Res. 2023;51:W587–92. 37144476 10.1093/nar/gkad359 PMC10320113

[43] Kropinski AM, Prangishvili D, Lavigne R. Position paper: the creation of a rational scheme for the nomenclature of viruses of *Bacteria* and *Archaea*. Environ Microbiol. 2009;11(11):2775–77. 19519870 10.1111/j.1462-2920.2009.01970.x

[44] Biebl H, Allgaier M, Tindall BJ, Koblizek M, Lünsdorf H, Pukall R, et al. *Dinoroseobacter shibae* gen. nov., sp. nov., a new aerobic phototrophic bacterium isolated from dinoflagellates. Int J Syst Evol Microbiol. 2005;55(3):1089–96. 15879238 10.1099/ijs.0.63511-0

[45] Rios AC, Moutinho CG, Pinto FC, Del Fiol FS, Jozala A, Chaud MV, et al. Alternatives to overcoming bacterial resistances: *state-of-the-art*. Microbiol Res. 2016;191:51–80. 27524653 10.1016/j.micres.2016.04.008

[46] Stojković EA, Rothman-Denes LB. Coliphage N4 *N*-Acetylmuramidase defines a new family of murein hydrolases. J Mol Biol. 2007;366:406–19. 17174325 10.1016/j.jmb.2006.11.028

[47] Moraru C, Varsani A, Kropinski AM. VIRIDIC—a novel tool to calculate the intergenomic similarities of prokaryote-infecting viruses. Viruses. 2020;12(11):1268. 33172115 10.3390/v12111268 PMC7694805

[48] Hutinet G, Kot W, Cui L, Hillebrand R, Balamkundu S, Gnanakalai S, et al. 7-Deazaguanine modifications protect phage DNA from host restriction systems. Nat Commun. 2019;10:5442. 31784519 10.1038/s41467-019-13384-y PMC6884629

[49] Huang X, Jiao N, Zhang R. The genomic content and context of auxiliary metabolic genes in roseophages. Environ Microbiol. 2021;23(7):3743–57. 33511765 10.1111/1462-2920.15412

[50] Shaburova OV, Krylov SV, Veiko VP, Pleteneva EA, Burkal’tseva MV, Miroshnikov KA, et al. Search for destruction factors of bacterial biofilms: comparison of phage properties in a group of *Pseudomonas putida* bacteriophages and specificity of their halo-formation products. Russ J Genet. 2009;45(2):161–70. 19334612

[51] Gallet R, Kannoly S, Wang I-N. Effects of bacteriophage traits on plaque formation. BMC Microbiol. 2011;11(1):181. 21827665 10.1186/1471-2180-11-181 PMC3176204

[52] Jin T, Yin J. Patterns of virus growth across the diversity of life. Integr Biol. 2021;13(2):44–59. 10.1093/intbio/zyab001 33616184

[53] Yang Y, Ma R, Yu C, Ye J, Chen X, Wang L et al. A novel *Alteromonas* phage lineage with a broad host range and small burst size. Microbiol Spectr. 2022;10(4). 10.1128/spectrum.01499-22 PMC9430817 35862972

[54] Storms ZJ, Sauvageau D. Modeling tailed bacteriophage adsorption: insight into mechanisms. Virology. 2015;485:355–62. 26331682 10.1016/j.virol.2015.08.007

[55] Dion MB, Oechslin F, Moineau S. Phage diversity, genomics and phylogeny. Nat Rev Microbiol. 2020;18(3):125–38. 32015529 10.1038/s41579-019-0311-5

[56] De Jonge PA, Nobrega FL, Brouns SJJ, Dutilh BE. Molecular and evolutionary determinants of bacteriophage host range. Trends Microbiol. 2019;27(1):51–63. 30181062 10.1016/j.tim.2018.08.006

[57] Zhan Y, Huang S, Voget S, Simon M, Chen F. A novel roseobacter phage possesses features of podoviruses, siphoviruses, prophages and gene transfer agents. Sci Rep. 2016;6:30372. 27460944 10.1038/srep30372 PMC4961962

[58] Zhang Y, Jiao N, Roseophage, RDJLΦ1. Infecting the aerobic anoxygenic phototrophic bacterium *Roseobacter denitrificans* OCh114. Appl Environ Microbiol. 2009;75(6):1745–49. 10.1128/AEM.02131-08 PMC2655476 19139231

[59] Nobrega FL, Vlot M, De Jonge PA, Dreesens LL, Beaumont HJE, Lavigne R, et al. Targeting mechanisms of tailed bacteriophages. Nat Rev Microbiol. 2018;16(12):760–73. 30104690 10.1038/s41579-018-0070-8

[60] Ignacio-Espinoza JC, Sullivan MB. Phylogenomics of T4 cyanophages: lateral gene transfer in the ‘core’ and origins of host genes. Environ Microbiol. 2012;14(8):2113–26. 22348436 10.1111/j.1462-2920.2012.02704.x

[61] Waldbauer JR, Coleman ML, Rizzo AI, Campbell KL, Lotus J, Zhang L. Nitrogen sourcing during viral infection of marine cyanobacteria. Proc Natl Acad Sci USA. 2019;116(31):15590–95. 31308237 10.1073/pnas.1901856116 PMC6681717

[62] Millard AD, Zwirglmaier K, Downey MJ, Mann NH, Scanlan DJ. Comparative genomics of marine cyanomyoviruses reveals the widespread occurrence of *Synechococcus* host genes localized to a hyperplastic region: implications for mechanisms of cyanophage evolution. Environ Microbiol. 2009;11(9):2370–87. 19508343 10.1111/j.1462-2920.2009.01966.x

[63] Gao S, Paez-Espino D, Li J, Ai H, Liang J, Luo Z, et al. Patterns and ecological drivers of viral communities in acid mine drainage sediments across Southern China. Nat Commun. 2022;13(1):2389. 35501347 10.1038/s41467-022-30049-5 PMC9061769

[64] Goldsmith DB, Crosti G, Dwivedi B, McDaniel LD, Varsani A, Suttle CA, et al. Development of *phoH* as a novel signature gene for assessing marine phage diversity. Appl Environ Microbiol. 2011;77(21):7730–39. 21926220 10.1128/AEM.05531-11 PMC3209181

[65] Yu Z, Li W, Ge C, Sun X, Wang J, Shen X, et al. Functional expansion of the natural inorganic phosphorus starvation response system in *Escherichia coli*. Biotechnol Adv. 2023;66:108154. 37062526 10.1016/j.biotechadv.2023.108154

[66] Nilsson E, Li K, Hoetzinger M, Holmfeldt K. Nutrient driven transcriptional changes during phage infection in an aquatic Gammaproteobacterium. Environ Microbiol. 2022;24(5):2270–81. 35049095 10.1111/1462-2920.15904 PMC9305737

[67] Zhu J, Yang F, Du K, Wei Z-L, Wu Q-F, Chen Y, et al. Phylogenomics of five *Pseudanabaena* cyanophages and evolutionary traces of horizontal gene transfer. Environ Microbiome. 2023;18(1):3. 36639816 10.1186/s40793-023-00461-5 PMC9837993

[68] Reader JS, Metzgar D, Schimmel P, De Crécy-Lagard V. Identification of four genes necessary for biosynthesis of the modified nucleoside queuosine. J Biol Chem. 2004;279(8):6280–85. 14660578 10.1074/jbc.M310858200

[69] Phillips G, El Yacoubi B, Lyons B, Alvarez S, Iwata-Reuyl D, De Crécy-Lagard V. Biosynthesis of 7-deazaguanosine-modified tRNA nucleosides: a new role for GTP cyclohydrolase I. J Bacteriol. 2008;190(24):7876–84. 18931107 10.1128/JB.00874-08 PMC2593212

[70] McCarty RM, Bandarian V. Biosynthesis of pyrrolopyrimidines. Bioorg Chem. 2012;43:15–25. 22382038 10.1016/j.bioorg.2012.01.001 PMC4022189

[71] Lanen SGV, Reader JS, Swairjo MA, Lee B, Iwata-Reuyl D, et al. From cyclohydrolase to oxidoreductase: Discovery of Nitrile reductase activity in a common Fold. PNAS. 2005;102(12):4264–69. 15767583 10.1073/pnas.0408056102 PMC555470

[72] Tsai R, Corrêa IR, Xu MY, Xu S. Restriction and modification of deoxyarchaeosine (dG+)-containing phage 9 g DNA. Sci Rep. 2017;7(1):8348. 28827753 10.1038/s41598-017-08864-4 PMC5567051

[73] Lang AS, Westbye AB, Beatty JT. The distribution, evolution, and roles of gene transfer agents in prokaryotic genetic exchange. Annu Rev Virol. 2017;4(1):87–104. 28784044 10.1146/annurev-virology-101416-041624

[74] Porse A, Gumpert H, Kubicek-Sutherland JZ, Karami N, Adlerberth I, Wold AE, et al. Genome dynamics of *Escherichia coli* during antibiotic treatment: transfer, loss, and persistence of genetic elements *in situ* of the infant gut. Front Cell Infect Microbiol. 2017;7:126. 28447026 10.3389/fcimb.2017.00126 PMC5388698

[75] Penadés JR, Chen J, Quiles-Puchalt N, Carpena N, Novick RP. Bacteriophage-mediated spread of bacterial virulence genes. Curr Opin Microbiol. 2015;(23):171–8. 10.1016/j.mib.2014.11.019 25528295

